# Retinal fundus imaging-based diabetic retinopathy classification using transfer learning and fennec fox optimization

**DOI:** 10.1016/j.mex.2025.103232

**Published:** 2025-02-17

**Authors:** Indresh Kumar Gupta, Shruti Patil, Supriya Mahadevkar, Ketan Kotecha, Awanish Kumar Mishra, Joel J.P.C. Rodrigues

**Affiliations:** a Pranveer Singh Institute of Technology, Kanpur, U.P, India; b Symbiosis Institute of Technology, Symbiosis International (Deemed University), Lavale, Pune, Maharashtra, India; c Symbiosis Centre for Applied Artificial Intelligence, Symbiosis International (Deemed University), Lavale, Pune, Maharashtra, India; d Maharana Pratap Engineering College, Kanpur, U.P, India; e COPELABS, Lusófona University, Lisbon, Portugal; f Federal University of Piauí (UFPI), Teresina, PI, Brazil

**Keywords:** Diabetic retinopathy, Fennec fox optimization, Deep learning, Inception-ResNet-v2, Median filter, Fundus Imaging Diabetic Retinopathy Classification using Deep Learning and Fennec Fox Optimization (FIDRC-DLFFO) model

## Abstract

Diabetic retinopathy (DR) is a serious complication of diabetes that can result in vision loss if untreated, often progressing silently without warning symptoms. Elevated blood glucose levels damage the retina's microvasculature, initiating the condition. Early detection through retinal fundus imaging, supported by timely analysis and treatment, is critical for managing DR effectively. However, manually inspecting these images is a labour-intensive and time-consuming process, making computer-aided diagnosis (CAD) systems invaluable in supporting ophthalmologists.

This research introduces the Fundus Imaging Diabetic Retinopathy Classification using Deep Learning and Fennec Fox Optimization (FIDRC-DLFFO) model, which automates the identification and classification of DR. The model integrates several advanced techniques to enhance performance and accuracy.

1. The proposed FIDRC-DLFFO model automates DR detection and classification by combining median filtering for noise reduction, Inception-ResNet-v2 for feature extraction, and a gated recurrent unit (GRU) for classification.
2. Fennec Fox Optimization (FFO) fine-tunes the GRU hyperparameters, boosting classification accuracy, with its effectiveness demonstrated on benchmark datasets.
3. The results provide insights into the model's effectiveness and potential for real-world application.

## Graphical abstract

**Figure:**
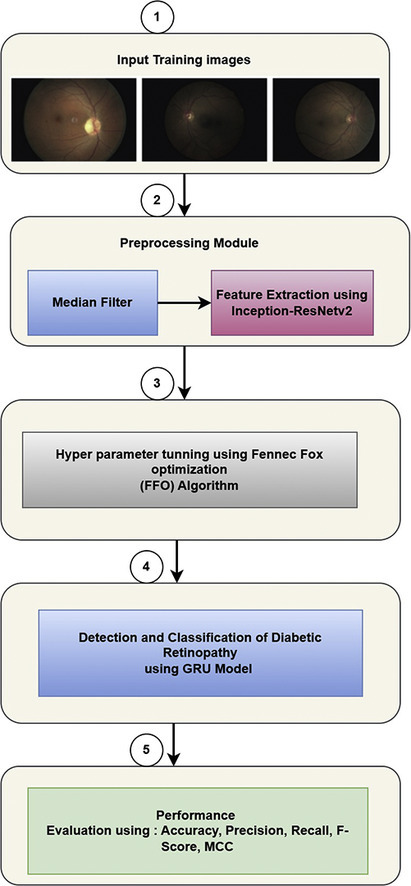

Specifications table

**Table:** 

Subject area:	Computer Science
More specific subject area:	Computer Vision- Fundus Imaging Diabetic Retinopathy
Name of your method:	Fundus Imaging Diabetic Retinopathy Classification using Deep Learning and Fennec Fox Optimization (FIDRC-DLFFO) model
Name and reference of original method:	NA
Resource availability:	Dataset Link- https://www.kaggle.com/c/diabetic-retinopathy-detection/discussion/234309.

## Introduction

Diabetes is an illness that arises once the pancreas doesn't excrete sufficient insulin, so the body is incapable of processing it well [1]. As diabetes evolves, the illness gradually impacts the circulatory system comprising the retina and arises because of prolonged accrued defaces to the blood vessels, decreasing the patient's vision and causing diabetic retinopathy (DR) [2]. In future, nearly 10 % of people lose their vision and about 2 % will experience extreme vision damage. As stated by an estimation by WHO, over 220 million people globally have diabetes [3]. DR is the main reason for vision loss in Type-2 diabetes patients and is categorized by chronic increasing injury of the retinal microvasculature. DR arises when blood glucose level affects the retinal blood vessels [4]. The upsurge of glucose levels in the blood can cause the arteries in the retina to deteriorate and drip into the eye, which causes blurred vision. During the next phase, the recently formed weaker blood vessels leak and break blood into the eye, resulting in blindness [5].

Screening a retina is a possible way to identify the damage produced in the retina in the early stages [6]. DR is asymptotic initially and consequently, many of the patients are unconscious of the illness unless it has emotional limitations on their vision. Consequently, consistent and prior screening of DR has been important to evade additional problems and to control the development of disease [7]. Deep learning (DL) based Convolutional Neural Network (CNN) was latterly proved to be a promising method for various medical image analysis, it offered effectual solutions to overwhelm the difficulties of gradient descent [8,9]. For additional processes, these mechanisms have been succeeded by image pre-processing and classifications for improving the precision of the next steps. The pre-processing of the image begins with the transformation of specified image features to a grayscale to perform further analysis [10]. The imaging classification stages aid in identifying the images based on the scheduled features, consequently receiving the anticipated outcomes. For the effective consideration of retinal disorders, imaging-based strategies using computer-aided diagnosis outperform traditional methods [11,12].

The primary innovations and significant contributions of this research work are mentioned below:

1. A novel Fundus Imaging Diabetic Retinopathy Classification using Deep Learning and Fennec Fox Optimization (FIDRC-DLFFO) model is developed.
2. This model incorporates median filter (MF) for effective noise reduction in the preprocessing stage.
3. It employs the Inception-ResNet-v2 method to perform feature extraction, enabling the identification of complex features associated with DR.
4. The FIDRC-DLFFO model utilizes a gated recurrent unit (GRU) technique for the classification of DR.
5. The Fennec Fox Optimization (FFO) optimally fine-tunes the hyperparameters of the GRU, resulting in enhanced classification performance.
6. The model has been validated through simulations using the Kaggle dataset, with outcomes evaluated based on various performance metrics.

## Background

Ather et al. [13] proposed a retinal vessel extraction method that surpassed existing models. Furthermore, anomalous images were classified. The fundus image datasets were interpreted by professionals for exudates and were later sent through a pre-processing phase for extraction. These were subsequently integrated into a single image and sent to two classifiers to categorize DR. Addanki and Sumathi [14] proposed an effective deep learning (DL) approach. The U-Net, as a key module, was employed for segmentation and was modified to make the methodology lightweight and cost-effective. Moreover, DRLeU-Net was assessed using an open dataset. The approach was also trained using the k-fold cross-validation model. Ramesh and Sathiamoorthy [15] introduced a novel dandelion optimization algorithm with a DL-based blood vessel segmentation and classification (DOADL-BVSC) technique for DR grading. This model combined the concepts of blood vessel segmentation and DL-based classification for DR diagnosis. To achieve this, the model utilized the fuzzy set type-II method for the image enhancement process. Subsequently, U-Net with a Bi-directional Feature Pyramid Network (U-BFPN) technique was used for effectively segmenting the blood vessels in retinal images. Furthermore, a squeeze and excitation (SE) model was implemented for feature vector generation with a DOA-based hyperparameter optimizer. Finally, the quantum autoencoder (QAE) method was used to detect and classify DR into separate phases.

Balasamy and Suganyadevi [16] focused on segmenting fundus image models by employing a fuzzy entropy multi-dimensional thresholding methodology. Additionally, an overall retinal image methodology was presented to encompass various discrepancies. These images collectively depicted the range of probable scenarios encountered by DR. Thomas and Jerome [17] assisted medical experts in predicting DR. The first step was to reduce noise and enhance image contrast. For noise mitigation, a Bilateral Filter (BF) and CLAHE model with an unsharp method were implemented. Moreover, the Extended Adaptive Density-based Spatial Clustering (EADSC) approach was used for segmentation. Ramasamy et al. [18] employed the adaptive NN-assisted Laplacian of Gaussian (AnLoG) classifying model on the extracted features to enhance DR disease diagnosis. The proposed methodology also implemented supervised BPN ML and other forecasting models.

## Method details

**Fig. 1:**
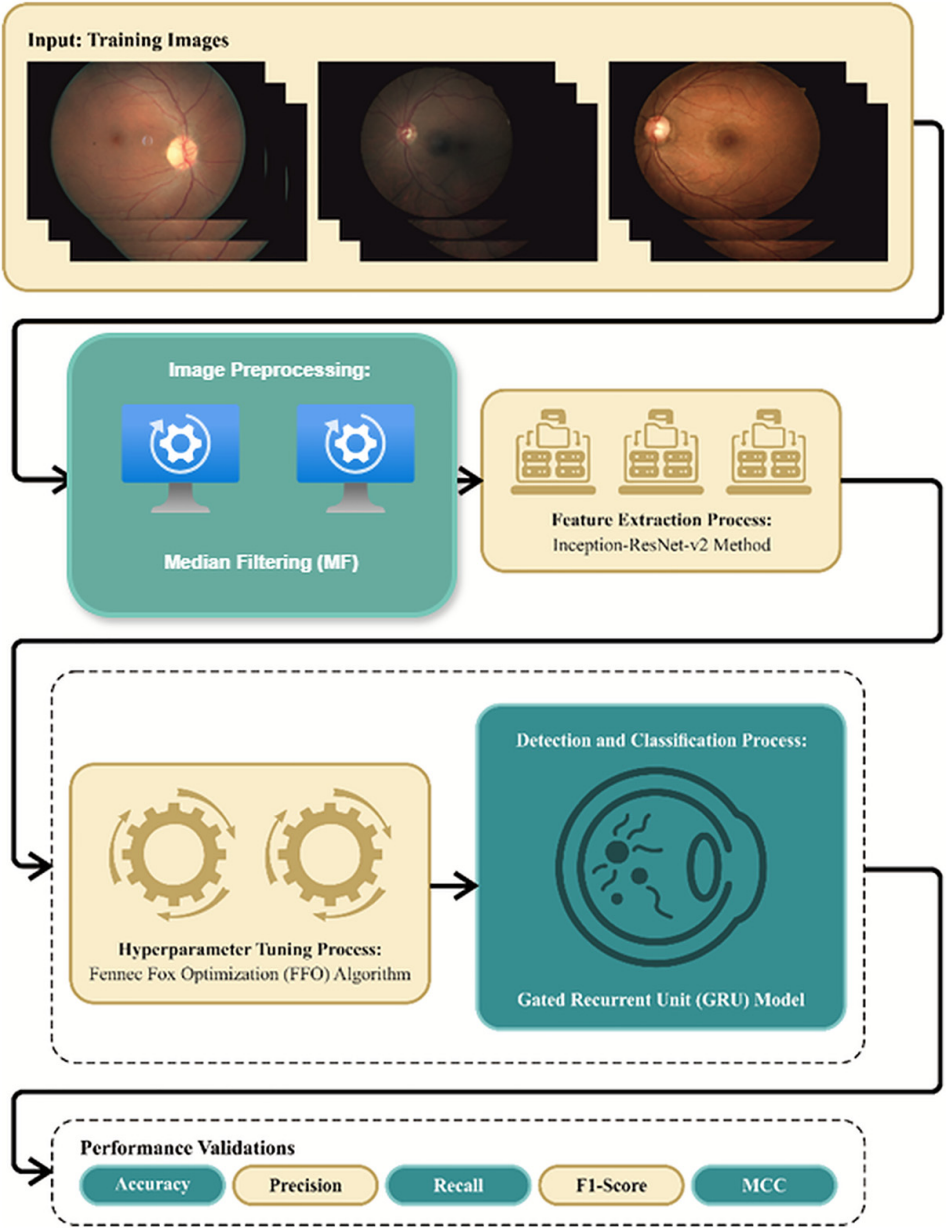
Overall process of FIDRC-DLFFO model.

This study offers the design of the FIDRC-DLFFO model. The presented FIDRC-DLFFO model automatically inspects RFIs for the identification and classification of DR. It comprises various processes, namely MF-based noise abatement, Inception-ResNet-v2-based feature extraction, GRU-based DR detection, and FFO-based parameter tuning, as shown in Fig. 1.

Stage I: Median filter for noise abatement

Image pre-processing enhances quality using a median filter (MF), which is a nonlinear technique that addresses noisy digital images by replacing each pixel with the median value derived from its surrounding pixels, referred to as a mask [19]. This operation involves sorting the pixels within the mask by their grayscale intensity values and substituting the noisy pixel with the calculated median from this group. The MF operation can be represented by g(x,y)=med{f(x−i,y−j),i,j∈W}, where *f*(*x,y*) *and* g(*x,y*) denotes the original and processed images respectively. The mask W is typically a two-dimensional array of size of n×n (e.g. 3×3,5×5, etc.) and can adopt various configurations, including linear, square, circular, or cross-shaped. The median filter's nonlinearity makes calculations more difficult, especially for images with random noise. The noise variance of the median filter for images with zero mean noise following a normal distribution is specified in Eq. (1).

(1) $$\sigma_{med}^{2}=\frac{1}{4nf^{2}\bar{\left(n\right)}}\approx \frac{\sigma_{i}^{2}}{n+\frac{\pi }{2}-1}\cdot \frac{\pi }{2}$$

The input power is $\sigma_{i}^{2}$ the median filter mask size is nand the noise density function is $f\bar{\left(n\right)}$. The noise effect on average filtering is given by Eq. (2).

(2) $$\sigma_{0}^{2}=\frac{1}{n}\sigma_{i}^{2}$$

Stage II: Inception-ResNet-V2 enabled feature extraction

Next, the FIDRC-DLFFO model uses the Inception-ResNet-v2 to extract features [20]. This method includes using its convolution layers to extract higher-level features.

Convolutional Layers: The Inception-ResNet-v2 model has a sophisticated architecture with multiple convolutional layers that detect and interpret crucial visual features at various abstraction levels and scales. To detect local features, the input image is subjected to several filters by each convolutional layer, aiming to extract features from the previous layer and create local connections. The convolutional operation applied to the input J is expressed using the filter $F\in R^{2x_{1}+2x_{2}}$ as shown in Eq. (3).

(3) $${\left(J*F\right)}_{m,n}=\sum_{g=-x_{1}}^{x_{1}}\sum_{i=-x_{2}}^{x_{2}}F_{\left(g,i\right)}J_{\left(m-g,n-i\right)}$$

The Filter F is given by Eq. (4).

(4) $$F=\left[\begin{array}{ccc} F_{-x_{1},-x_{2}} & \cdots & F_{-x_{1},x_{2}}\\ \vdots & F_{0,0} & \vdots \\ F_{x_{1},-x_{2}} & \cdots & F_{x_{1},x_{2}} \end{array}\right]$$

The feature map uses ReLU as its activation function.

Hierarchical Feature Representation: After the pre-processed RFIs are feed to the Inception-ResNet-v2 model, they are processed in order through the convolutional layers. Each layer extracts features that become progressively more complex and abstract. The lower layers prioritize basic elements such as edges, angles, and textures, in contrast, the higher layers recognize more complex forms and patterns.

Inception-ResNet-v2 Architecture: The Inception-ResNet-v2 model integrates inception modules that enable feature extraction at multiple scales by using parallel convolutional paths with varying filter dimensions, effectively capturing features across various receptive fields. Residual connections are employed to mitigate the vanishing gradient issue, enhancing deep network training. By leveraging diverse scales and convolution kernels, the model improves adaptability while simplifying overall complexity and speeding up parameter optimization. Additionally, 1 × 1 convolutions adjust feature map dimensions, as the feature map $y_{h}$ may vary in the residual convolution network. The residual operation is detailed in Eqs. (5) to (7).

(5) $$F\left(y_{h}\right)=e*+\beta$$

(6) $$x_{h}=Rl\left(F\right)+s\left(y_{h}\right)$$

(7) $$y_{h+1}=Rl\left(x_{h}\right)$$

In this scenario βindicates the offset, $y_{h}$ stands for the input, and $x_{h}$ represents the combined result from the two branches. The weight is denoted by e and Rl symbolizes the ReLU activation function. The convolutional operation is expressed as $F(y_{h}),$with $y_{h+1}$ representing the ultimate output of the residual module. Furthermore, $s(y_{h})$illustrates the straightforward transformation applied to the input. The ReLU activation function is defined by Eq. (8).

(8) Rl(y)=max(0,y)

By using y and a threshold of 0 as inputs, the output can be calculated, addressing the vanishing gradient problem during Inception network training. This is a key goal of the residual network learning unit, enabling faster and simpler training when saturation is reached. It is defined by Eq. (9).

(9) $$\frac{\alpha Ym}{\alpha Yj}=\frac{\alpha Yj+F\left(Yj,\tau j,\beta j\right)}{\alpha Yj}=1+\frac{\alpha F\left(Ym,\tau m.\beta m\right)}{\alpha Ym}$$

where Yj represents the input of the j−th residual unit, while Ym denotes the input of *the* m*-th* residual unit, with the residual function indicated as F(.).

Feature Map Extraction: During each convolutional layer, the model creates a series of feature maps, often referred to as activation maps. Each of these maps illustrates the response of a specific filter to the input image, highlighting areas that contain certain features or patterns. As the network goes deeper, the feature maps reflect progressively higher levels of abstraction.

Dimensionality Reduction: To enhance efficient feature representation and reduce computational complexity, the Inception-ResNet-v2 model employs dimensionality reduction techniques. This is typically accomplished using methods like 1 × 1 pooling operation. The feature maps' spatial dimensions are reduced while maintaining key features using dimensionality reduction.

Stage III: Gated recurrent units based classification

For the classification process, the FIDRC-DLFFO model is implemented using the GRU framework [21]. The key failures in the output circuit of the signal conditioning component are due to ongoing stress effects, and these failures are dependent on time. Consequently, predicting circuit module failures is reframed as a time-series forecasting challenge. Recurrent Neural Networks (RNNs) possess short-term memory capabilities and find widespread application in time-series prediction tasks. However, conventional RNNs face challenges such as exploding gradients, vanishing gradients, and limited ability to process long-range data features.

We suggest using Long Short-Term Memory (LSTM) networks to overcome these difficulties. In order to overcome the drawbacks of conventional recurrent neural networks (RNNs), LSTMs use gating methods to increase their memory capacity. However, there are some disadvantages to LSTMs as well, namely the need for more parameters and longer training periods.

A less complex kind of Long Short-Term Memory (LSTM) network is called a Gated Recurrent Unit (GRU). One update gate is created by combining the input and forget gates. Efficiency is increased with this modification. In addition to tracking the neurons' memory, GRUs also significantly speed up the training process. The core elements of the GRU architecture include the reset gate $r_{t}$ and the update gate $z_{t}$. The working principle of a GRU system is described by Eqs. (10)-(13).

(10) $$r_{t}=\sigma \left(W_{r}\times x_{t}+U_{r}\times h_{t-1}+b_{r}\right)$$

(11) $$z_{t}=\sigma \left(W_{z}\times x_{t}+U_{z}\times h_{t-1}+b_{z}\right)$$

(12) $${\hat{h}}_{t}=\text{tanh}\left(W_{h}\times x_{t}+r_{t}\times U_{h}\times h_{t-1}+b_{h}\right)$$

(13) $$h_{t}=\left(1-z_{t}\right)\times h_{t-1}+z_{t}\times {\hat{h}}_{t}$$

In this context, $x_{t}$ denotes to the input at the current time step, while $h_{t}$ indicates the output produced during that same time frame. The term $h_{t-1}$ signifies the output from the prior time step. The value $r_{t}$ represents the activation result from the reset gate, and $z_{t}$ denotes the output from the update gate activation. Additionally, ${\hat{h}}_{t}$ corresponds to the candidate hidden state at the current time step. The symbols W and U are used for the weight matrices, while b represents the bias term.

Stage IV: Fennec fox optimization driven hyperparameter tuning

The hyperparameter tuning process of the FIDRC-DLFFO model is accomplished using the FFO [22]. The fennec fox, the smallest canid species, is distinguished by its large ears, straw-colored fur, and fur-covered paw pads suited for the sandy Sahara and Sinai Peninsula. Its unique ear structure helps adapt to north african deserts. The FFO is inspired by two key fennec fox strategies: digging to find prey and escaping predators, which form the foundation of its mathematical model. Primarily the population of fennec fox can be initialized at random based on the limit of these questions, and the actual process is shown by Eq. (14).

(14) $$X_{i}:x_{ij}=lb_{j}+r.\left(Ub_{j}-lb_{j}\right)\;with\;i=1,2,\ldots \ldots M;\;j=1,2,\ldots \ldots N$$

where $X_{i}$ denotes the i−th fennec fox, the control variable is $x_{ij}$, which denotes the i−th fennec fox in the j−th dimension. The number of control variables is denoted by N, while the total number of fennec foxes is indicated by M. Within the range [0,1], the random variabler is limited. Furthermore, $lb_{j}$ and $Ub_{j}$ indicate the control variable's lower and upper limits, respectively.

The position of FFO members in the search space is updated using two natural behaviors of the fennec fox: (i) digging to capture prey hidden under the sand and (ii) evading predators.

### Digging to capture prey hidden under the SAND

The fennec fox hunts alone at night, using its large ears to locate prey under the sand, then digs to catch it. This local search behaviour enhances FFO's exploitation ability to find solutions near the global optimum. A neighbourhood radius R around its position models this digging behavior, allowing convergence to better solutions. This phase of updating the FFO members is mathematically represented using Eqs. (15)-(17).

(15) $$x_{i,j}^{\left(P_{1}\right)}=x_{i,j}+\left(2\times r-1\right)\times R_{i,j}$$

(16) $$R_{i,j}=\alpha \times \left(1-\frac{1}{T}\right)\times x_{i,j}$$

(17) $$X_{i}=\left\{ \begin{array}{cc} X_{i}^{\left(P_{1}\right)} & ifF_{i}^{\left(P_{1}\right)}<F_{i}\\ X_{i} & otherwise \end{array}\right.$$

where, the newly proposed state of the fennec fox based on the first phase is denoted by $X_{i}^{\left(P_{1}\right)}$, its j−th dimension is denoted by $x_{i,j}^{\left(P_{1}\right)}$, and its objective function value is represented by $F_{i}^{\left(P_{1}\right)}$. Furthermore, the neighbourhood radius for $x_{i,j}$ is indicated by $R_{i,j}$the counter for iterations ist, the total number of iterations is indicated by T, and α is a constant set to 0.2.

### Evading predators

The fennec fox faces threats from predators such as caracals, striped hyenas, and pharaoh eagle-owls, but it effectively evades them with its remarkable speed and agility. This escape mechanism inspires a mathematical model aimed at global exploration in the search space. By simulating this behaviour, the FFO strategy enhances exploration capabilities, allowing for avoidance of local optima and easing the search for the global optimum. As a result, the random positioning of potential solutions within the search space mirrors the fennec fox's evasion tactics. This phase of updating the FFO members is mathematically represented using Eqs. (18)-(20).

(18) $$X_{i}^{rand}:x_{i,j}^{rand}=x_{k,j}\;with\;K=1,2,\ldots \ldots ,M;i=1,2,\ldots \ldots \ldots M$$

(19) $$x_{i,j}^{\left(P_{2}\right)}=\left\{ \begin{array}{cc} x_{i,j}+r\times \left(x_{i,j}^{rand}-I\times x_{i,j}\right) & F_{i}^{rand}<F_{i}\\ x_{i,j}+r\times \left(x_{i,j}-x_{i,j}^{rand}\right) & otherwise \end{array}\right.$$

(20) $$X_{i}=\left\{ \begin{array}{cc} X_{i}^{\left(P_{2}\right)} & ifF_{i}^{\left(P_{2}\right)}<F_{i}\\ X_{i}Y & otherwise \end{array}\right.$$

where, the target position that the fennec fox in the i−thdimension seeks to reach during its escape is represented by $X_{i}^{rand}$, its position in the j−th dimension is indicated by $x_{i,j}^{rand}$, and its objective function value is denoted by $F_{i}^{rand}$. Additionally, $X_{i}^{\left(P_{2}\right)}$ is the updated state of the i−th fennec fox based on the second phase, with $F_{i}^{\left(P_{2}\right)}$ as its objective function value and $x_{i,j}^{\left(P_{2}\right)}$ denoting its position in the j−th dimension. Finally, the number I is selected at random from the collection {1, 2}.

The FFO utilizes a fitness function (FF) to enhance classification performance, where FF is represented by "Fitness." In this study, classification accuracy serves as the FF, with the primary goal of maximizing this function. The FF is defined in Eq. (21).

(21) $$\text{Fitness}=\frac{\text{Number}\;\text{of}\;\text{Accurately}\;\text{Classified}\;\text{Images}}{\text{Total}\;\text{Image}\;\text{Count}}\times 100$$

## Method validation

**Fig. 2:**
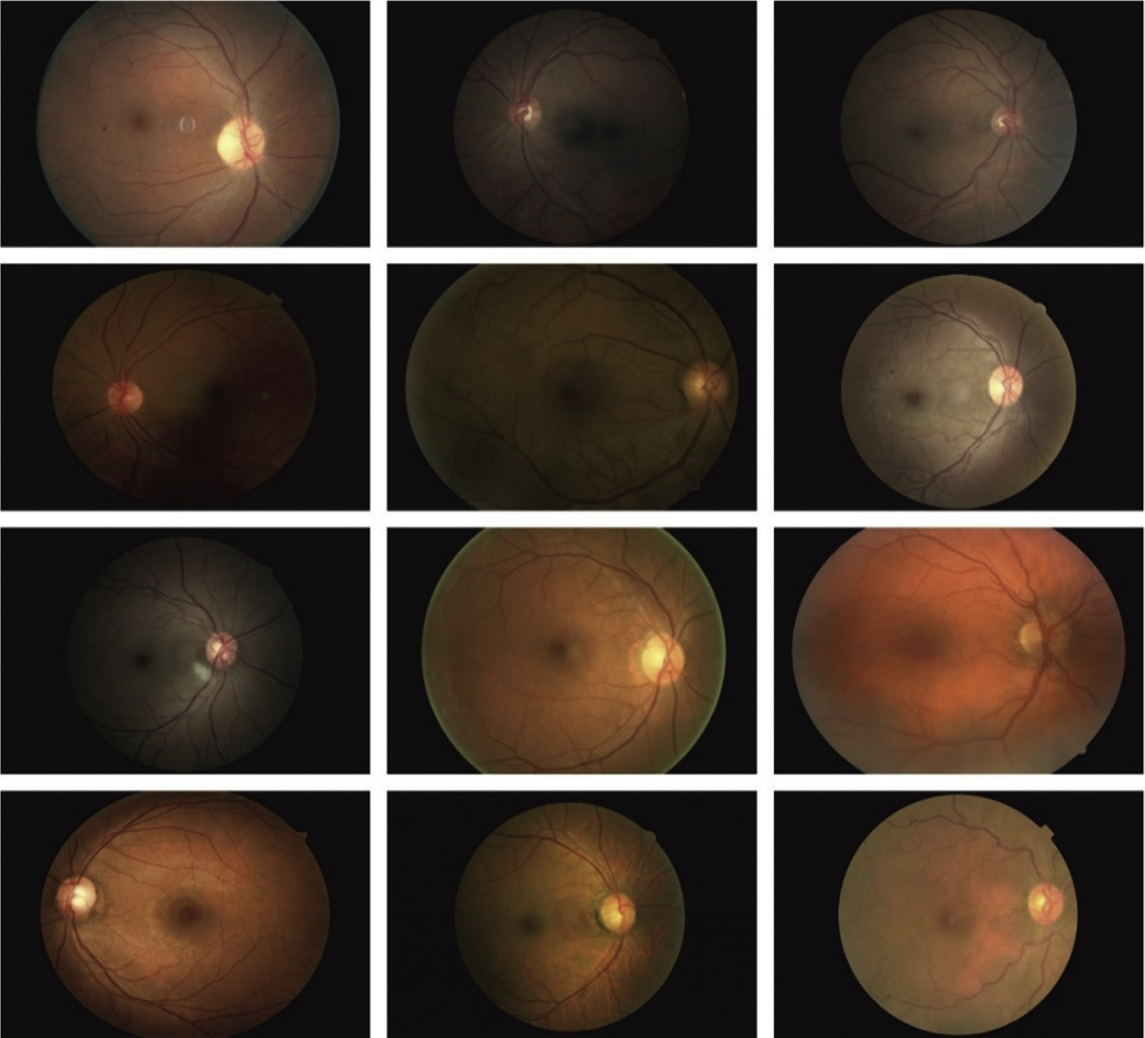
Sample images.

**Fig. 3:**
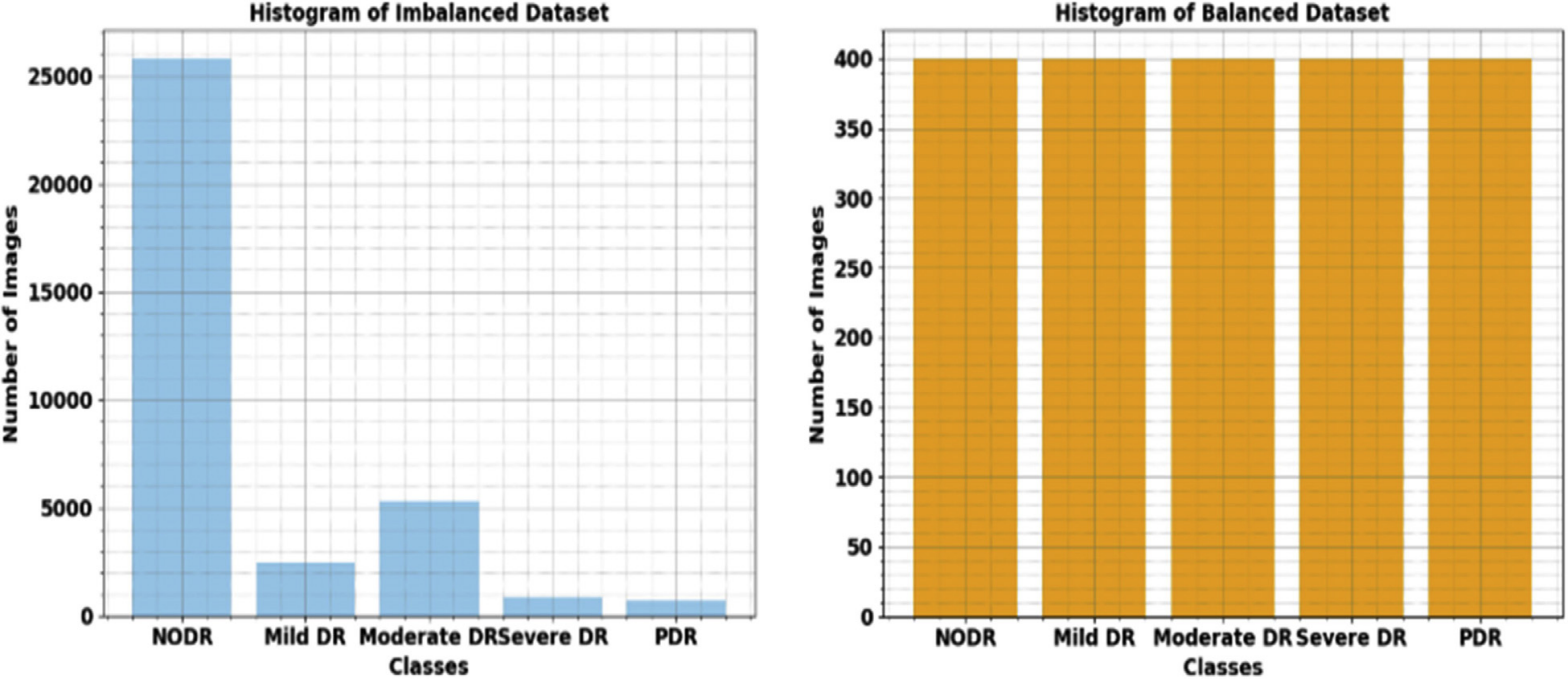
Histogram of dataset images.

To evaluate the performance of the FIDRC-DLFFO model on a Kaggle dataset [23] that includes five classes of DR: Mild DR (2443 images), Moderate DR (5292 images), severe DR (873 images), PDR (708 images), and NODR (25,810 images) used. Fig. 2 represents the sample images. To guarantee a balanced dataset, 400 samples chosen randomly from each class for this research. It mitigates the effect of class imbalance to boost classification effectiveness. Fig. 3 depicts the histogram before and after data balanced.

**Fig. 4:**
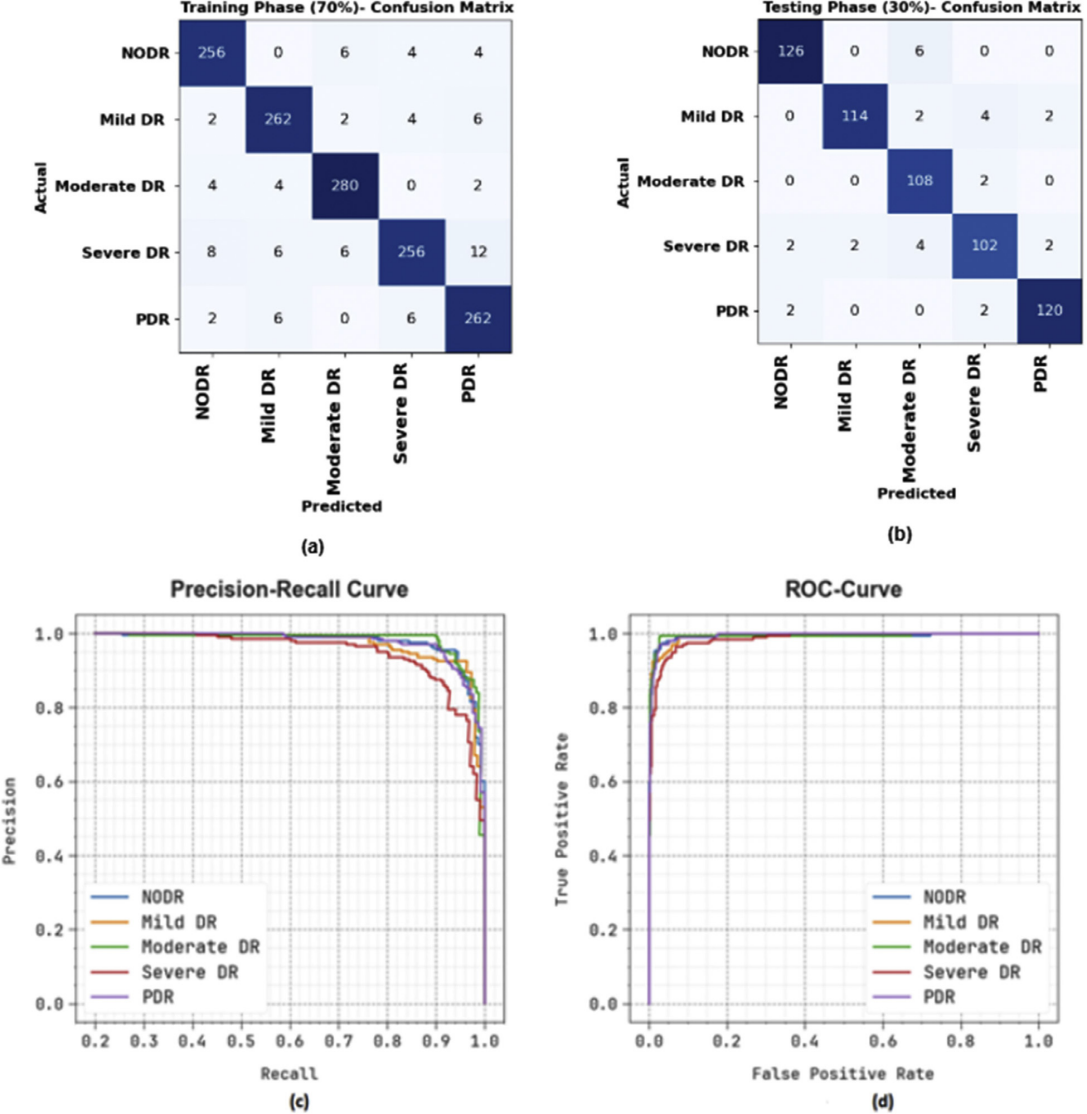
Classification outcomes: (a-b) Confusion matrices and (c-d) PR and ROC curves.

Fig. 4 displays the classification outcomes of the FIDRC-DLFFO model, demonstrating its ability to accurately detect and classify data. Fig. 4(a) and 4(b) show confusion matrices for the 70 % training partition (TRAP) and 30 % testing partition (TESP), highlighting the model's high accuracy with minimal misclassification. Fig. 4(c) presents precision-recall (PR) analysis, confirming the model's strong performance across all classes, particularly in handling imbalanced data. The PR curve indicates strong precision and recall values, crucial for minimizing false positives and negatives. Fig. 4(d) shows the receiver operating characteristic (ROC) curve, with high area under the curve (AUC) values, reflecting effective class discrimination and an optimal balance between sensitivity and specificity. These results collectively highlight the FIDRC-DLFFO model's robustness and suitability for real-world classification tasks.

**Fig. 5:**
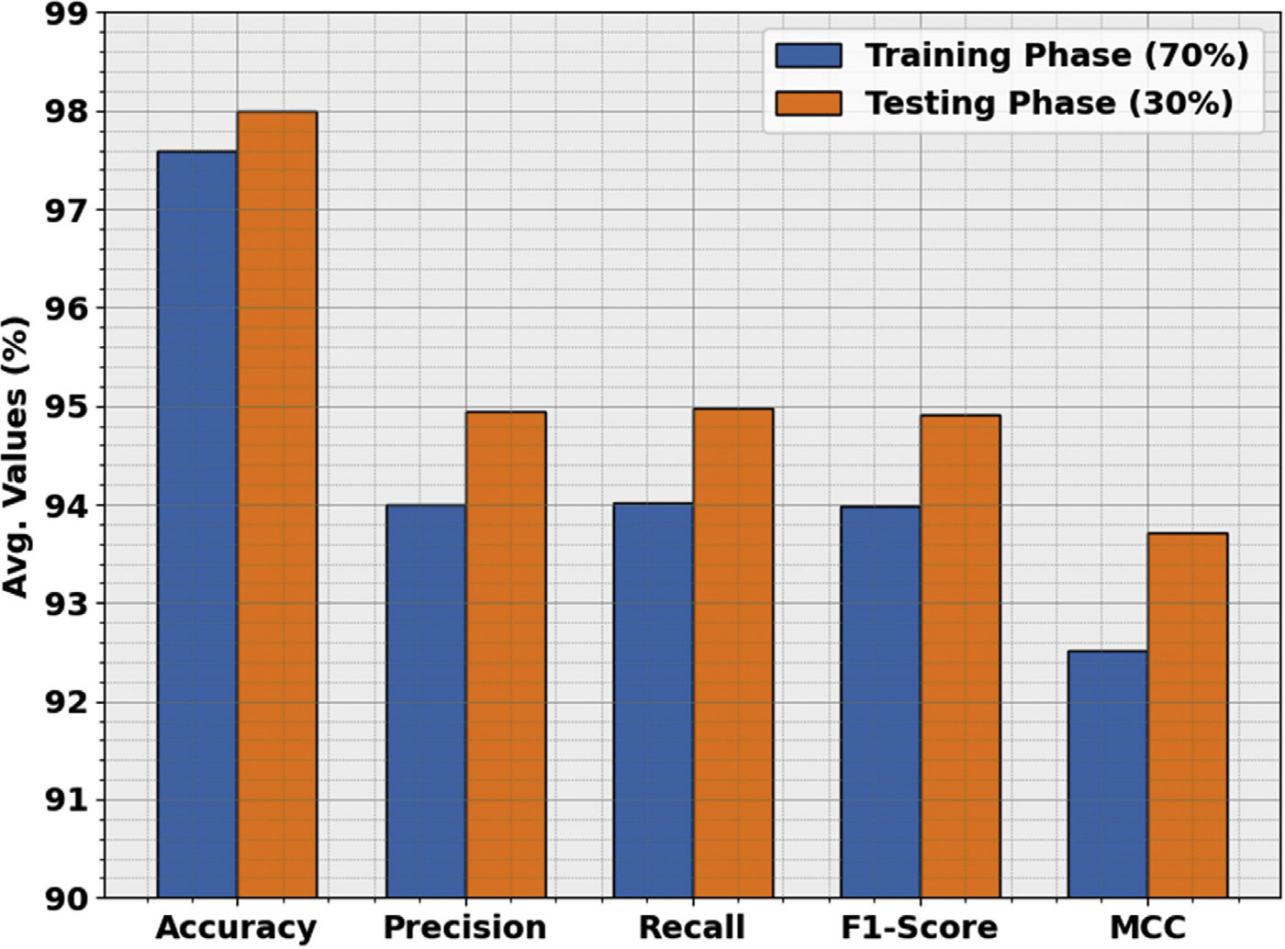
Result analysis of FIDRC-DLFFO model with 70 % TRAP and 30 % TESP.

Table 1 and Fig. 5 assess the FIDRC-DLFFO model's performance with a 70 % TRAP and 30 % TESP. The model demonstrates strong classification accuracy across all classes. With 70 % TRAP, the model achieves average scores of 97.60 % accuracy, 94.00 % precision, 94.02 % recall, 93.99 % F1-score, and 92.51 % MCC. On the 30 % TESP, it slightly improves, attaining 98.00 % accuracy, 94.94 % precision, 94.98 % recall, 94.91 % F1-score, and 93.71 % MCC. The increase in performance on the testing set indicates the model's ability to generalize well to unseen data. These high values across all metrics highlight the model's reliability, making it suitable for real-world classification tasks and ensuring its effectiveness across different data subsets.

**Table 1:** Result analysis of FIDRC-DLFFO model with 70 % TRAP and 30 % TESP.

Class	Accuracy	Precision	Recall	F1-Score	MCC
**TRAP (70 %)**					
**NODR**	97.86	94.12	94.81	94.46	93.14
**Mild DR**	97.86	94.24	94.93	94.58	93.25
**Moderate DR**	98.29	95.24	96.55	95.89	94.81
**Severe DR**	96.71	94.81	88.89	91.76	89.78
**PDR**	97.29	91.61	94.93	93.24	91.56
**Average**	**97.60**	**94.00**	**94.02**	**93.99**	**92.51**
**TESP (30 %)**					
**NODR**	98.33	96.92	95.45	96.18	95.12
**Mild DR**	98.33	98.28	93.44	95.80	94.81
**Moderate DR**	97.67	90.00	98.18	93.91	92.61
**Severe DR**	97.00	92.73	91.07	91.89	90.06
**PDR**	98.67	96.77	96.77	96.77	95.71
**Average**	**98.00**	**94.94**	**94.98**	**94.91**	**93.71**

**Fig. 6:**
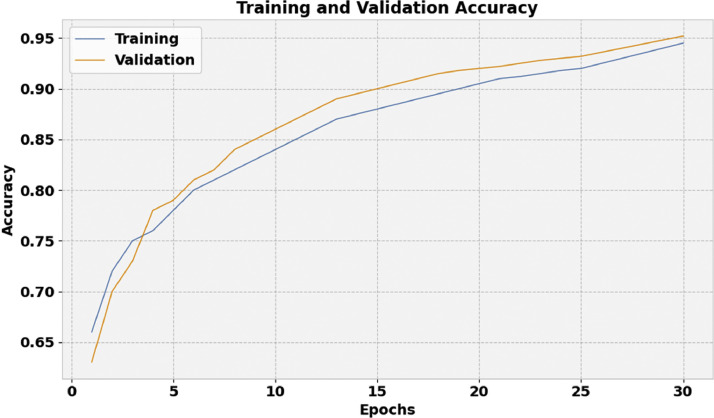
Accuracy curve of the FIDRC-DLFFO model.

Fig. 6 depicts the training accuracy (TRA) and validation accuracy (VLA) curve of the FIDRC-DLFFO model. The accuracy values are computed over a range of 0–30 epochs. The results demonstrate the ability of the FIDRC-DLFFO model to enhance performance over several iterations by showing a rising trend in both TRA and VLA values. Additionally, the model's capacity to produce accurate predictions on unknown data is reinforced by the strong alignment of TRA and VLA across epochs, which suggests minimal overfitting.

**Fig. 7:**
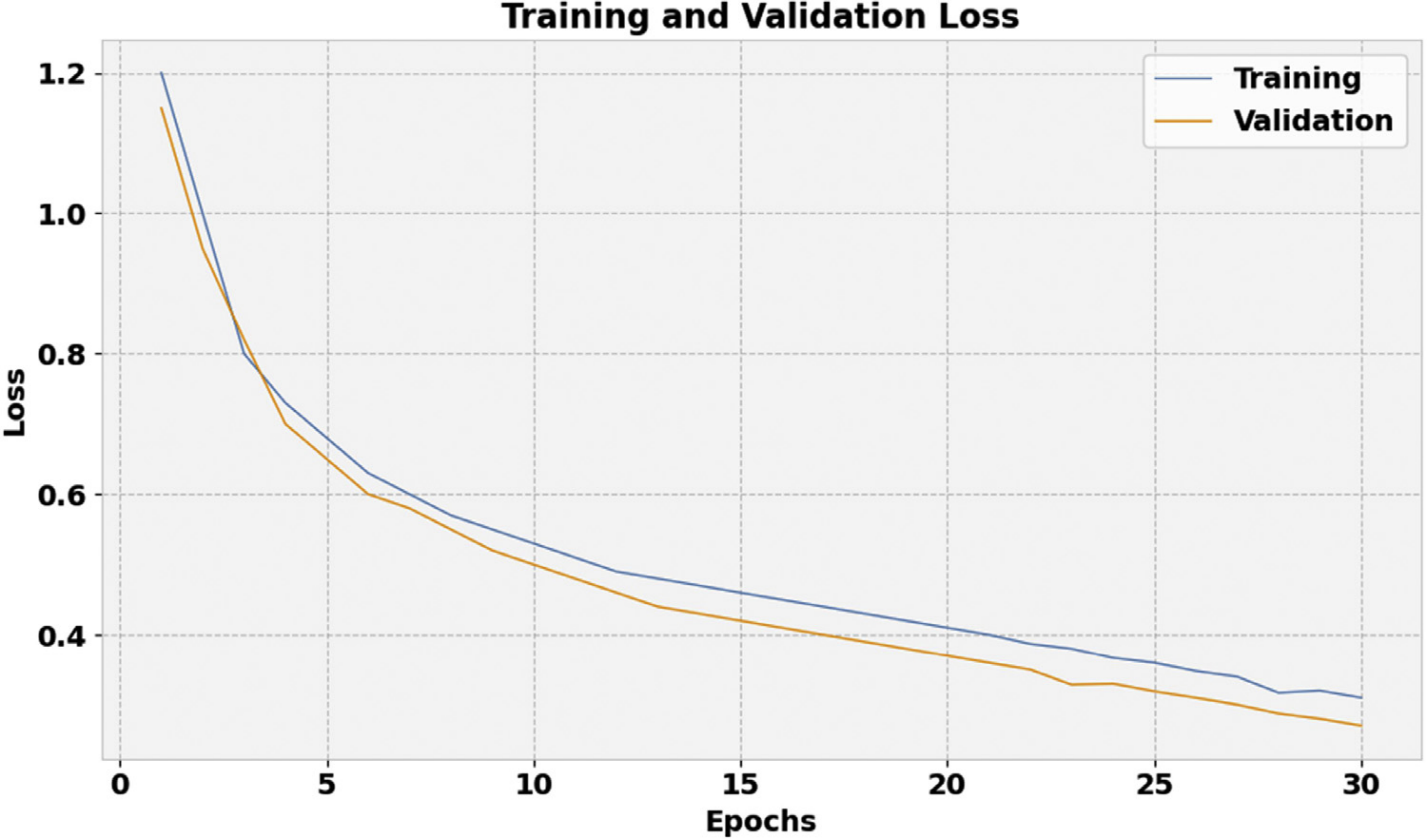
Loss curve of the FIDRC-DLFFO model.

Fig. 7 depicts the training loss (TRAL) and validation loss (VLAL) curve of the FIDRC-DLFFO model. The loss values are computed over a range of 0–30 epochs. Both TRAL and VLAL values exhibit a declining trend in the results, indicating that the FIDRC-DLFFO model successfully strikes a balance between fitting the data and generalization. This steady decline in loss values emphasizes the model's enhanced performance even more, which eventually produces better predicted results.

**Fig. 8:**
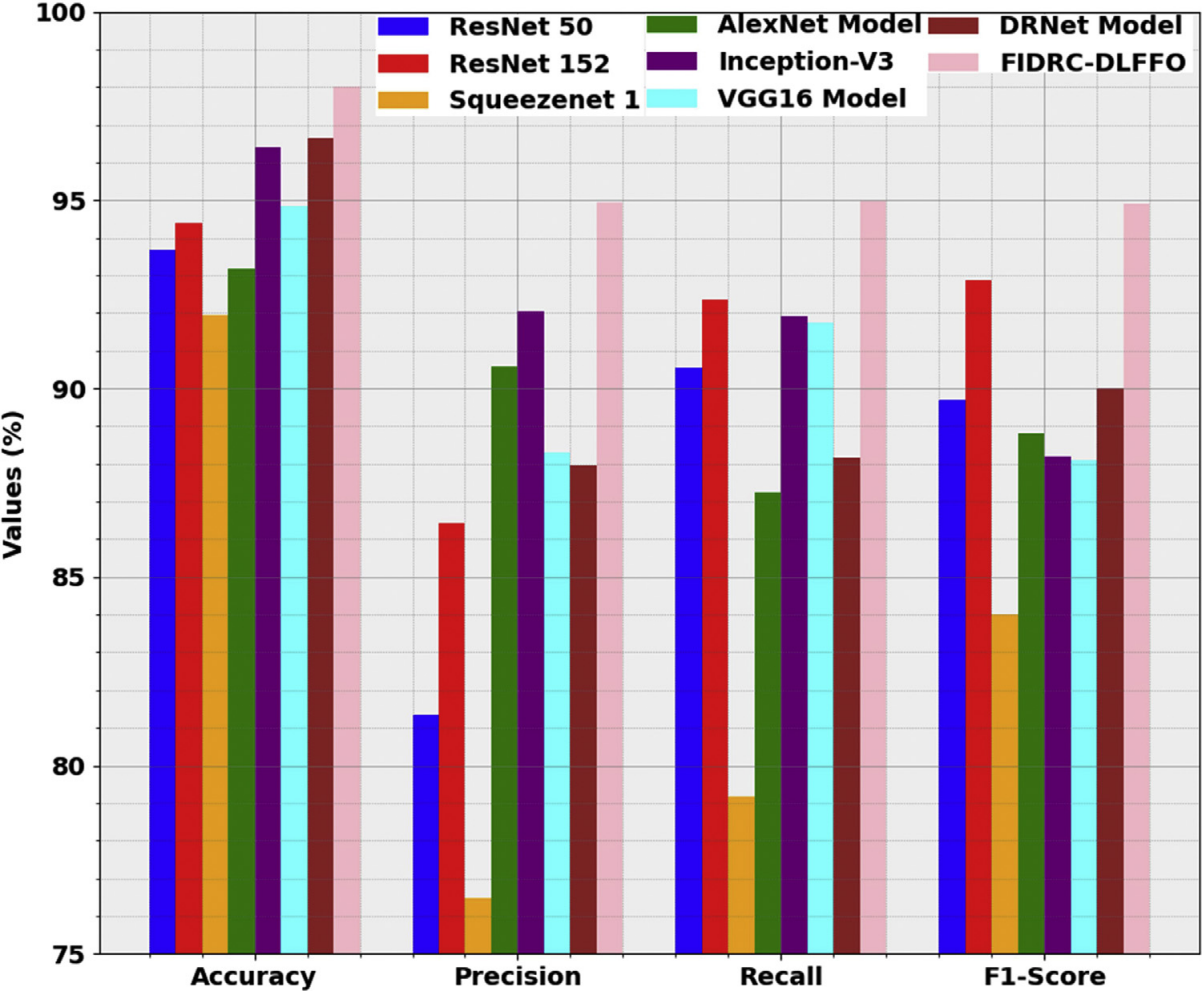
Comparative analysis of the FIDRC-DLFFO model with recent models.

Table 2 and Fig. 8 compare the performance of the FIDRC-DLFFO model with other existing models, highlighting its superior effectiveness. The results show that models like ResNet 50, ResNet 152, Squeezenet 1, AlexNet, and VGG16 performed less effectively, with lower accuracy, precision, recall, and F1 scores. Inception-V3 and DRNet yielded better results but still fell short of FIDRC-DLFFO's performance. The FIDRC-DLFFO model achieved an impressive 98.00 % accuracy, 94.94 % precision, 94.98 % recall, and 94.91 % F1- score, surpassing the other models in all key metrics. This demonstrates the model's superior ability to identify relevant instances while maintaining a balanced and reliable performance, making it a strong contender in the field. Overall, the FIDRC-DLFFO model sets a new standard for performance in classification tasks.

**Table 2:** Comparative analysis of the FIDRC-DLFFO model with recent models.

Classification Model	Accuracy	Precision	Recall	F1-Score
ResNet 50	93.67	81.35	90.53	89.68
ResNet 152	94.40	86.41	92.35	92.87
Squeezenet 1	91.94	76.47	79.17	84.01
AlexNet Model	93.18	90.58	87.24	88.80
Inception-V3	96.40	92.06	91.91	88.20
VGG16 Model	94.83	88.31	91.75	88.10
DRNet Model	96.65	87.96	88.15	90.01
**FIDRC-DLFFO**	**98.00**	**94.94**	**94.98**	**94.91**

## Conclusion

This research work has presented the design of the FIDRC-DLFFO model. The presented FIDRC-DLFFO model has automatically inspected RFIs for the detection and classification of DR. Initially, the FIDRC-DLFFO model has applied image preprocessing using the MF for noise reduction. Following that, the Inception-ResNet-v2 method has been used to perform feature extraction, identifying complex features and patterns related to DR. For the DR classification, the FIDRC-DLFFO model has employed the GRU technique. Finally, the FFO algorithm has fine-tuned the hyperparameter outcomes of the GRU technique optimally, resulting in better classification performance. The FIDRC-DLFFO model has been validated through simulations using a Kaggle dataset, with its performance evaluated across various metrics. The results provide insights into the model's effectiveness and potential for real-world application.

## Limitations

Not applicable.

## CRediT authorship contribution statement

**Indresh Kumar Gupta:** Validation, Writing – review & editing, Writing – original draft. **Shruti Patil:** Conceptualization, Methodology, Software. **Supriya Mahadevkar:** Validation, Writing – review & editing, Writing – original draft. **Ketan Kotecha:** Supervision. **Awanish Kumar Mishra:** Visualization, Investigation. **Joel J.P.C. Rodrigues:** Software, Validation.

## Declaration of competing interest

The authors declare that they have no known competing financial interests or personal relationships that could have appeared to influence the work reported in this paper.

## Data Availability

Datalink is mentioned in the manuscript.
